# Drug affinity and targeted delivery: double functionalization of silk spheres for controlled doxorubicin delivery into Her2-positive cancer cells

**DOI:** 10.1186/s12951-020-00609-2

**Published:** 2020-03-30

**Authors:** Kamil Kucharczyk, Anna Florczak, Tomasz Deptuch, Karolina Penderecka, Katarzyna Jastrzebska, Andrzej Mackiewicz, Hanna Dams-Kozlowska

**Affiliations:** 1 Chair of Medical Biotechnology, Poznan University of Medical Sciences, 61-688 Poznan, Poland; 2 Department of Diagnostics and Cancer Immunology, Greater Poland Cancer Centre, 15 Garbary St, 61-688 Poznan, Poland

**Keywords:** Silk, Genetic engineering, Spheres, Drug carrier, Doxorubicin, Cancer

## Abstract

**Background:**

The optimal drug delivery system should be biocompatible, biodegradable, and allow the sustained release of the drug only after it reaches the target cells. Silk, as a natural polymer, is a great candidate for building drug carriers. Genetically engineered silks offer the possibility of functionalization. Previously, we characterized bioengineered silk spheres that were functionalized with H2.1 peptide that selectively delivered a drug to Her2-positive cancer cells. However, drug leakage from the silk spheres showed the need for improved control.

**Results:**

To control the drug loading and release, we designed and produced functional silk (DOXMS2) that contains a DOX peptide with an affinity for doxorubicin. The DOXMS2 spheres showed the decreased release of doxorubicin compared with MS2 particles. Next, the DOXMS2 silk was blended with the H2.1MS1 polymer to improve the control of doxorubicin binding and release into Her2-positive cancer cells. The H2.1MS1:DOXMS2 particles showed the highest doxorubicin-loading capacity and binding per cell, which resulted in the highest cytotoxic effect compared with that of other sphere variants. Since drug release at a pH of 7.4 from the blended H2.1MS1:DOXMS2 particles was significantly lower than from blended spheres without DOXMS2 silk, this indicated that such particles could control the release of the drug into the circulatory system before the carrier reached the tumor site.

**Conclusions:**

This strategy, which is based on the blending of silks, allows for the generation of particles that deliver drugs in a controlled manner.

## Background

The protocols for breast cancer treatment combine surgery, radiotherapy, and chemotherapy. Chemotherapeutics are known for inducing toxic effects after their systemic administration. Therefore, potent anticancer drugs, such as doxorubicin, which show good results in the treatment of tumors, are associated with some risk. The nondiscriminating biodistribution of doxorubicin may lead to severe cardiomyopathy and heart failure, among other side effects [1]. The application of drug delivery systems should reduce the adverse effects associated with this drug.

Many doxorubicin delivery systems for breast cancer treatment have been proposed [2]. They vary in terms of their physicochemical properties and pharmacokinetics and the materials used for the production of the vehicle. They also employ different strategies for drug delivery (i.e., active or passive drug delivery). Doxil^®^ and Myocet^®^ are the two formulations that have already been approved by the US Food and Drug Administration (FDA) and are prominently used in both breast and ovarian cancer treatment. Although liposome-based doxorubicin delivery systems significantly reduce the adverse effects of anticancer therapy in comparison with free doxorubicin, many patients still experience severe side effects [3]. Both Myocet^®^ and Doxil^®^ deliver doxorubicin to the tumor site due to the enhanced permeability and retention (EPR) effect. The EPR effect allows the accumulation of small drugs and nanoparticles in the tumor environment due to fenestrations in the vascular system and impaired drainage of the lymphatic system [4]. Many of the currently investigated drug-carrying systems take advantage of this passive approach to drug delivery [5]. The employment of active targeted delivery should significantly reduce possible unwanted side effects associated with off-target cytotoxicity. An example of such an approach utilizes immunoliposomes for Her2-positive breast cancer treatment [6]. The incorporation of an anti-Her2 antibody fragment into the liposome vehicle led to the targeted delivery of the incorporated drug in in vitro and in vivo studies [6].

Another shortcoming of drug delivery systems is the fast clearance of vehicles from the circulation. Although modifications of the liposomes, such as PEGylation (for example, in Doxil^®^), can extend the carrier half-life in the circulation, there is still much room for improvement. Moreover, drug loading efficiency and drug release kinetics are crucial aspects of the design of drug delivery systems. The efficient encapsulation of drugs can contribute to the increased efficacy of the therapy by reducing the required dose for the formulation. Depending on the material used for the production of the vehicle and the method of drug incorporation, the drug encapsulation efficiency may significantly vary between systems [7]. For instance, depending on the technique used for doxorubicin incorporation in chitosan-based drug delivery systems, the entrapment efficiency of the drug in the vehicle varied from 19 to 97.2% [7]. Furthermore, drug delivery systems should also allow sustained release of the drug only after reaching the targeted destination and without drug leakage into the circulatory system.

Natural polymers have gained much attention as materials used to build drug carriers. Although natural polymers do not offer as much control over the structure and physical properties as synthetic materials, they provide biocompatibility and biodegradability. For the production of doxorubicin drug delivery carriers, natural polymers such as chitosan, silk, or hyaluronic acid have been investigated in in vitro studies [7]. Silk is a natural polymer with excellent mechanical properties, biocompatibility, and biodegradability [8]. Moreover, silk proteins have been used to form various morphological structures, such as sponges, nonwoven mats, hydrogels, films, fibers, scaffolds, capsules, and spheres [8–10]. Predominantly, silk biomaterials are made of silkworm silk extracted from *Bombyx mori* cocoons or spider silks that are mainly biotechnologically produced [9, 11]. The bioengineered spider silks are synthetic proteins with amino acid sequences similar to their natural equivalents [12]. Based on the amino acid sequence of natural silk, the oligonucleotides are designed, synthesized, and then used as monomeric ‘building blocks.’ After their multimerization by ligation, the obtained artificial gene encodes polymeric silk [12]. The bioengineered silk proteins are produced in a heterologous host, and after the purification process, they can be used for the formation of biomaterials that possess a desired morphological structure [12, 13].

The materials are often functionalized to better address the needs of the desired application. As mentioned above, bioengineered spider silks are encoded by synthetic genes that are constructed at the DNA level, which provides the opportunity to introduce a DNA sequence that encodes a functional peptide, domain or other protein [14–16]. Hybrid (chimeric) silk materials functionalized with binding domains have been reported to efficiently bind nucleic acids, small drugs or proteins [14]. Furthermore, silk polymers have also been successfully modified with motifs possessing targeting properties towards specific cells [17–19].

In our previous work, we designed and characterized the bioengineered spider silk proteins MS1 and MS2, which were designed based on major ampullate spidroin 1 and major ampullate spidroin 2, respectively, from the spider *N. clavipes* [20]. The MS1 and MS2 proteins were functionalized with H2.1 peptide, which binds to Her2 [21]. Spheres made of H2.1MS1 protein selectively delivered the drug to Her2-positive cancer cells in in vitro studies [17]. In this study, we designed and produced a different functional silk—DOXMS2. The recombinant DOXMS2 silk was obtained through the introduction of an oligonucleotide encoding the DOX peptide into the cDNA sequence of MS2. As a result, a new functional property was introduced into the MS2 protein, as the DOX peptide possesses affinity towards doxorubicin [22]. The designed DOXMS2 protein was then blended with the H2.1MS1 protein to improve the control of binding and the release of doxorubicin into Her2-positive cancer cells. The H2.1MS1:DOXMS2 spheres were characterized in terms of morphology, drug binding and release, and the ability to selectively deliver Dox to the target cells.

## Results and discussion

Spider silk has been considered a material with great potential for drug delivery applications. As mentioned earlier, an effective drug carrier should meet specific requirements. It should ensure delivery of the drug to the site of action, ultimately reducing the amount of drug administered to the patient. Moreover, the carrier material should protect the drug from degradation, efficiently encapsulate the drug, and control the drug release. In our previous work, to address these issues and obtain a smart drug delivery system for cancer therapies, we applied different approaches. To control the properties of silk spheres, we functionalized the silk protein with peptides, modified the silk amino acid sequence, established a silk purification procedure, blended two different bioengineered spider silk proteins, and implemented highly controllable and repeatable automatic conditions for the sphere formation process [17, 21, 23–25]. However, better control over the drug loading capacity and sustained release properties of the silk carriers were still required. The drug release process should avoid burst release into the bloodstream before the carrier can reach the tumor, and the effect of the drug on tumor cells should be prolonged.

In the present study, we examined a new strategy for obtaining silk particles with improved drug loading and release capabilities. We designed a novel variant of bioengineered silk (DOXMS2) that contained a peptide with affinity towards doxorubicin. First, the DOXMS2 spheres were characterized in terms of size, morphology, and loading/release capacity, and their properties were compared with the properties of the MS2 particles.

### Construction, production, and purification of bioengineered silks

**Fig. 1:**
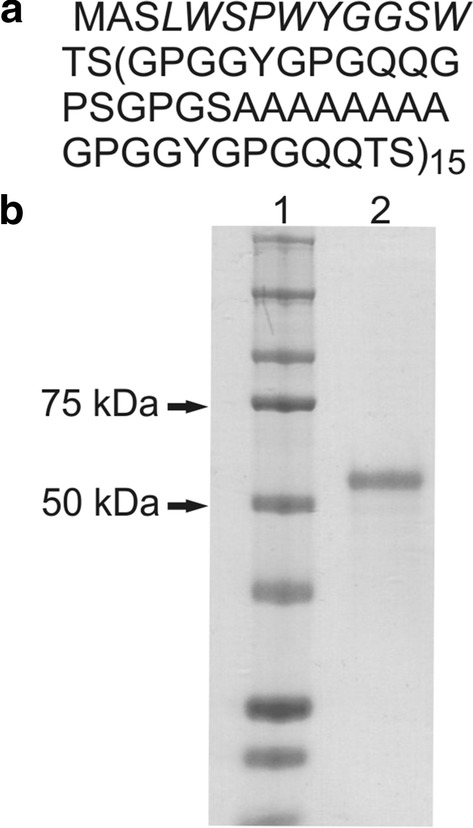
Analysis of the bioengineered DOXMS2 silk. **a** The amino acid sequence of the DOXMS2 construct. The protein consists of 15 repeats of a monomeric unit derived from MaSp2 silk from the spider *N. clavipes* and a peptide DOX with affinity towards doxorubicin (indicated in italics). **b** SDS-PAGE analysis of purified DOXMS2. *1* Precision Plus Protein ™ Kaleidoscope ™ molecular weight marker; *2* DOXMS2 silk

The sequence of the novel functional protein DOXMS2 is indicated in Fig. 1a. As mentioned above, the DOXMS2 protein contains a peptide (DOX) that has an affinity towards doxorubicin (Dox). The oligonucleotide encoding DOX was fused to the sequence of the bioengineered spider silk protein MS2. The SDS-PAGE analysis indicated that the purified protein was free from impurities and did not degrade (Fig. 1b). According to the gel analysis, the molecular weight of DOXMS2 did not correspond to the expected weight (48.129 kDa). Our previous data concerning the MS2 protein indicated that although the SDS-PAGE analysis also showed a higher than expected molecular weight for the silk protein, the MALDI-TOF results were in agreement with the predicted value [24]. Similarly, the migration of DOXMS2 in the SDS-PAGE gel was impaired.

The quality of other silks used in the next steps of the study, such as MS2, MS1, and H2.1MS1, were determined previously [17, 20].

### Morphology and size of plain DOXMS2 and MS2 spheres

**Fig. 2:**
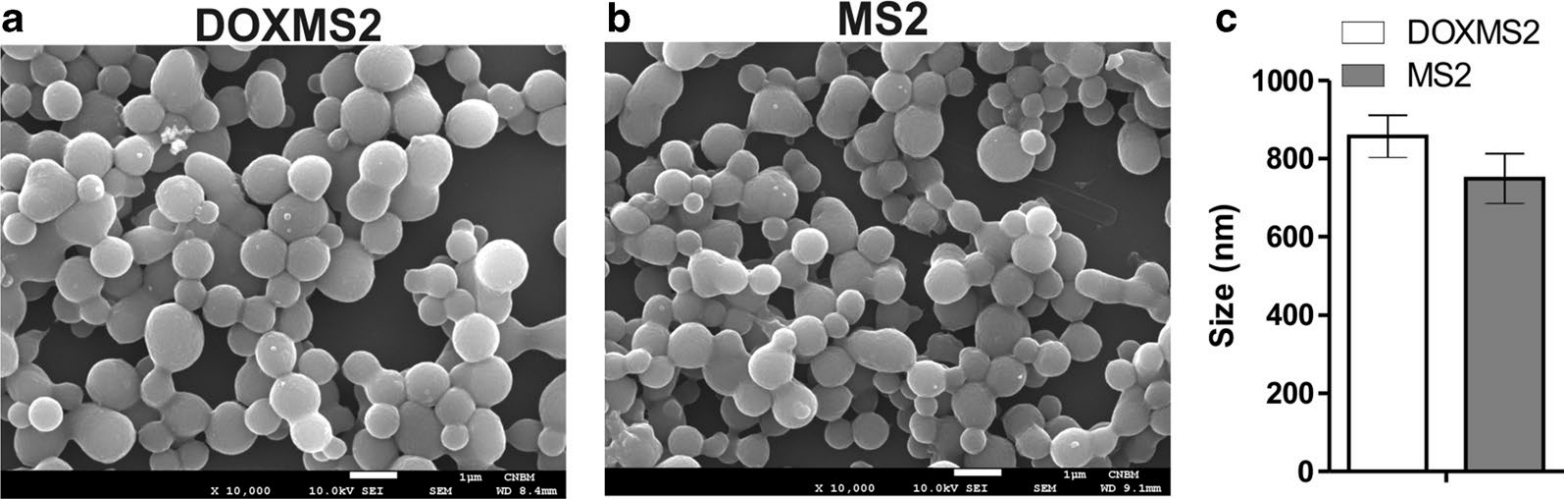
Morphology and size of DOXMS2 and MS2 spheres. SEM analysis of **a** DOXMS2 and **b** MS2 particles that were formed by mixing silk at an initial concentration of 2.5 mg/mL with 2 M potassium phosphate buffer, pH 8. Scale bar—1 µm. **c** The mean size of the spheres was calculated by measuring the diameter of 100 particles in SEM images. The mean and standard deviation of three independent experiments are shown

Two variants of the plain silk spheres were produced: (1) DOXMS2 and (2) MS2. The SEM analysis demonstrated that both the DOXMS2 and MS2 silk proteins formed particles with a spherical morphology (Fig. 2a, b). The mean diameter of the DOXMS2 spheres was slightly larger than that of the MS2 particles; however, the difference was not significant (Fig. 2c). The DOX peptide did not affect the self-assembly properties of MS2 silk and the process of sphere formation; similar results were reported previously for different functional peptides [21, 26].

### Doxorubicin loading and release: analysis of the plain DOXMS2 and MS2 spheres

**Fig. 3:**
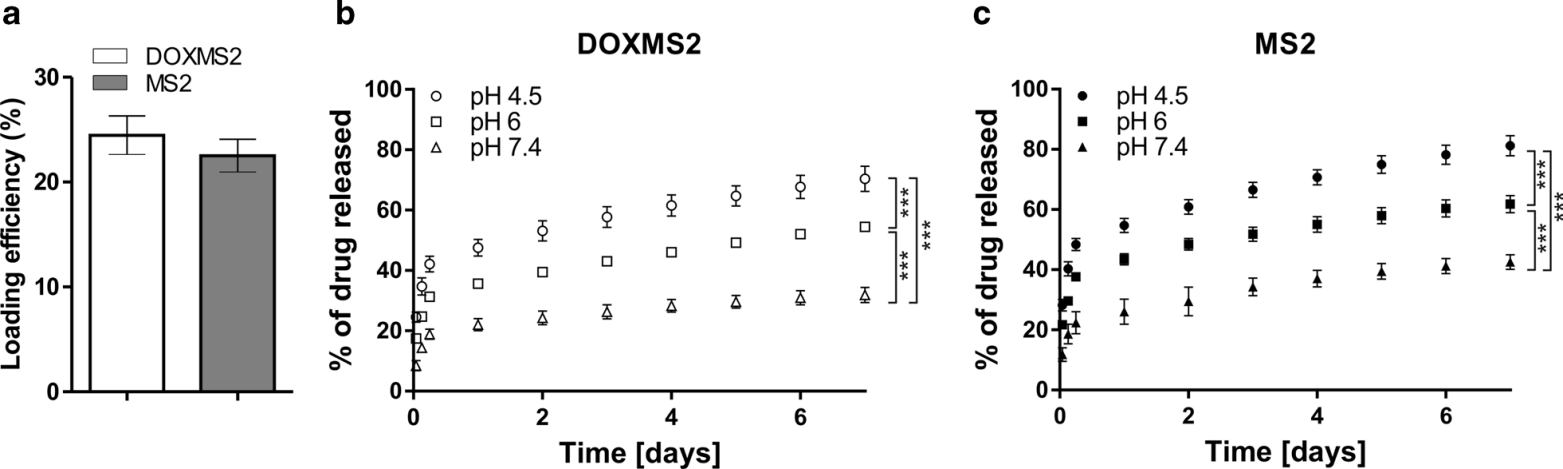
The loading efficiency and release kinetics of doxorubicin from plain silk spheres. **a** The postloading method was used for the incorporation of Dox into spheres. Dox was released from **b** DOXMS2 and **c** MS2 particles. The loaded spheres were resuspended in PBS buffer at pH 7.4, 6 or 4.5 and incubated at 37 °C for 7 days. At the indicated time points, the amount of released drug was evaluated spectrophotometrically. During the first day, the Dox release was determined after 1, 3 and 6 h of incubation. *** indicates statistical significance with p < 0.001

Drug loading into spheres was performed using the postloading method described previously [20]. A slightly higher efficiency of Dox loading into spheres functionalized with DOX peptide was observed compared to that of MS2 particles. However, the difference was not significant (Fig. 3a).

The release of doxorubicin was performed over 7 days of incubation at 37 °C in phosphate-buffered saline at pH 7.4, 6, and 4.5 (Fig. 3b, c). Independent of the variation of the examined spheres, Dox demonstrated a pH-dependent release profile, and the highest release efficiency was observed at a pH of 4.5 and the lowest at a pH of 7.4. These data were in agreement with our previous results concerning the interactions between doxorubicin and various types of silk at different pH values [20, 25]. This was due to the higher solubility of protonated Dox [27] and the weaker interactions between Dox and silk particles at lower pH values. The pH-dependent release of Dox has also been proposed for many other delivery systems [28–34]. In general, the slower Dox release from silk spheres at pH 7.4 than at 4.5 might be beneficial for increasing Dox stability in the circulatory system and enhancing Dox release in the tumor microenvironment.

The DOXMS2 spheres exhibited less effective release of doxorubicin in comparison with the MS2 particles (Fig. 3b, c). After 3 h of incubation, the DOXMS2 spheres released approximately 22%, 16%, and 14% less Dox at pH 7.4, 6 and 4.5, respectively, than the MS2 particles. After 7 days, the release of Dox from the DOXMS2 spheres was 23% less at pH 7.4, approximately 12% less at pH 6 and 4.5 than that from the MS2 particles (Fig. 3b, c). Although the incorporation of the DOX peptide did not significantly increase Dox loading, it allowed for significantly improved control of Dox release.

### Blended spheres

Although the DOXMS2 spheres displayed a more beneficial release of Dox than the MS2 spheres, these spheres would not be fully effective in releasing the drug to the target cells, i.e., cancer cells. The DOXMS2 carriers do not possess the correct tag (address) that would allow the specific delivery of the drug carrier. Such particles could be only useful for passive drug delivery that utilizes the EPR effect. To overcome this issue and to develop a system for effective active targeted drug delivery, herein, we employed the controlled blending of two different bioengineered silk proteins: (i) H2.1MS1 silk, which contains the H2.1 peptide that recognizes the Her2 molecule, and (ii) DOXMS2 silk, which shows an affinity for Dox. As mentioned above, we previously investigated the targeted drug delivery system by using H2.1MS1 spheres in a breast cancer model [17]. We found that the H2.1MS1 silk spheres were specifically bound and internalized into Her2-overexpressing cells. Moreover, we previously indicated that the plain MS1 and MS2 spheres differed in terms of their properties [20]. MS2 particles presented better characteristics in terms of morphology and colloidal stability than MS1 spheres. Unfortunately, the plain MS2 particles functionalized with the H2.1 peptide did not efficiently deliver Dox into Her2-overexpressing cells [21]. To resolve this problem, we established a strategy of sphere formation by blending functionalized MS1 and MS2 silks at a ratio of 8:2 to form particles that bound to the target cells at the same level as the functionalized MS1 spheres but had greatly improved physicochemical properties [21]. By blending two H2.1 functionalized silks (MS1 and MS2), we specifically combined the advantageous characteristics of both types of silk spheres: gained specificity, improved stability and enhanced efficiency of sphere formation [21]. Thus, in this study, we applied a similar strategy and blended two silks, H2.1MS1 and DOXMS2, to form drug delivery vehicles with specific targeting properties and the improved loading/release of doxorubicin, respectively.

### The morphology and size of the blended spheres

**Fig. 4:**
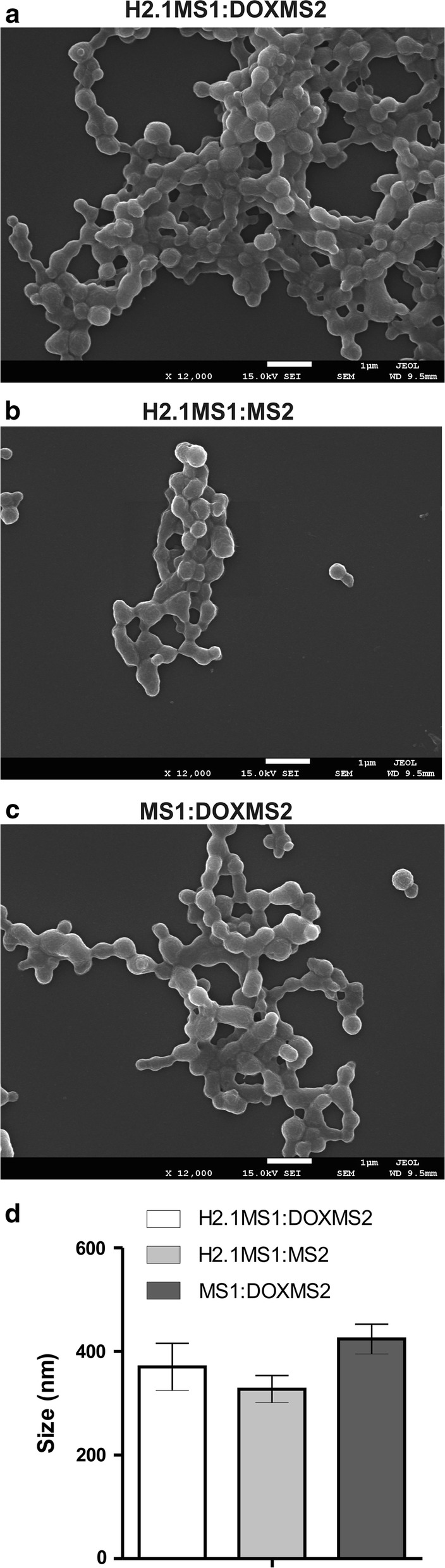
SEM images and size of blended spheres. **a** H2.1MS1:MS2, **b** H2.1MS1:DOXMS2 and **c** MS1:DOXMS2 particles were formed by blending the indicated silks at a volume ratio of 8:2, and then silk solutions with an initial concentration of 0.5 mg/mL were mixed with 2 M potassium phosphate buffer, pH 8, using high-pressure syringe pumps. Scale bar—1 µm. **d** The mean size of the spheres was determined by the analysis of the diameter of 100 particles in SEM images. The mean and standard deviation of three independent experiments are shown

The silks MS1, H2.1MS1, MS2, and DOXMS2 were used to produce the following blended spheres as indicated: (1) H2.1MS1:DOXMS2, (2) H2.1MS1:MS2, and (3) MS1:DOXMS2. First, the corresponding silks were mixed at a ratio of 8:2. Then, the spheres were produced by mixing the silk solution with potassium phosphate buffer using high-pressure syringe pumps. The sphere variants containing the MS1 type of silk exhibited a less well defined morphology than particles made of the plain MS2 type of silk (Fig. 4 vs. 2). This was in agreement with our previous data; the MS2 protein formed well-defined spherical particles, while the MS1 and H2.1MS1 silks formed less spherical, more aggregated particles [17, 20]. As expected, the blended sphere variants were smaller than plain spheres (Fig. 4 vs. 2) because a lower concentration of silk solution was used for their production. The size of the spheres depended on the concentration of the silk protein used; as the silk concentration increased, the size of the particles increased [20, 24, 25, 35, 36]. Moreover, the size of the H2.1MS1:DOXMS2 particles was slightly larger than that of the H2.1MS1:MS2 spheres, and the MS1:DOXMS2 spheres had the largest mean diameter; however, these differences were not significant (Fig. 4d).

### Doxorubicin loading and release: the analysis of blended spheres

**Fig. 5:**
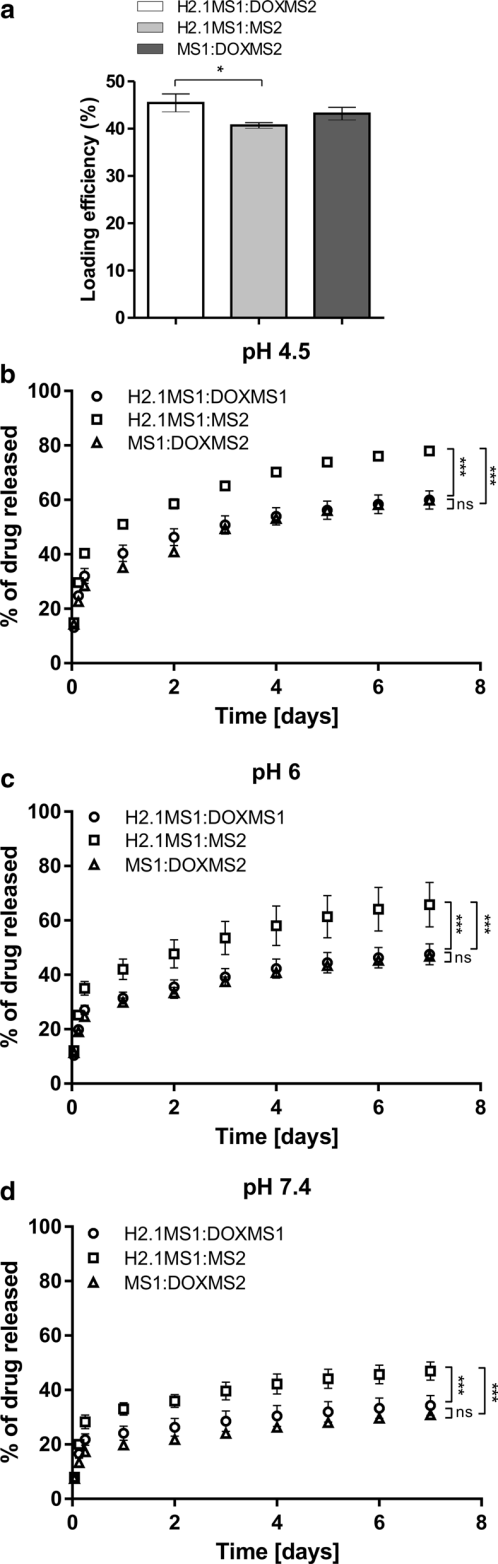
The loading efficiency and release kinetics of doxorubicin from the blended silk spheres. **a** A postloading method was used for Dox incorporation into spheres. The mean and standard deviation of three experiments are shown. Dox was released from **b** H2.1MS1:DOXMS2, **c** H2.1MS1:MS2, and **d** MS1:DOXMS2 particles. The Dox-loaded spheres were resuspended in PBS buffer at pH 7.4, 6 and 4.5 and incubated at 37 °C for 7 days. At the indicated time points, the amount of released drug was measured spectrophotometrically. During the first day, the Dox release was determined after 1, 3, and 6 h of incubation. The experiment was performed in triplicate. * indicates statistical significance with p < 0.05; *** indicates statistical significance with p < 0.001

The incorporation of Dox into H2.1MS1:DOXMS2, H2.1MS1:MS2, and MS1:DOXMS2 spheres and the release study were performed as described above. The loading efficiency of Dox into the H2.1MS1:DOXMS2 spheres was significantly 12% higher than that of Dox into the H2.1MS1:MS2 particles and was slightly higher compared with that of loading into the MS1:DOXMS2 spheres (Fig. 5a). This confirmed the role of the DOX peptide in enhancing the loading efficiency of Dox.

For each variant of the blended silk spheres, a pH-dependent release profile of Dox was generated that showed the acceleration of Dox release at an acidic pH (Fig. 5b–d), similar to plain spheres (Fig. 3b, c). The blended spheres that contained the DOXMS2 silk revealed slower drug release profiles than spheres without DOX peptide at all tested pH values (Fig. 5b–d). The release of Dox from the spheres that contained DOX peptide was approximately 23%, 28%, and 30% lower at pH 4.5, 6, and 7.4, respectively, compared with the release from the H2.1MS1:MS2 spheres (Fig. 5b–d). Dox release from the H2.1MS1:DOXMS2 and MS1:DOXMS2 particles was similar (Fig. 5b–d).

An optimal drug carrier should be capable of releasing an active agent in a controlled way so that the therapeutic payload is released only at the destination site. As indicated in the present study, the Dox release profile differed considerably between the blended particles containing DOX peptide and the blended variant spheres with control MS2 silk. Our previous results showed that spheres made of MS1 silk incorporated more Dox than plain MS2 spheres [20]. On the other hand, MS2 spheres showed a lower Dox release rate than the MS1 particles at a pH of 7.4 [20]. By blending the H2.1MS1 and H2.1MS2 silks, we obtained spheres that efficiently loaded Dox, delivered Dox into Her2-overexpressing cancer cells, and released decreased amounts of Dox at a pH of 7.4 [21]. In this study, by incorporating DOX peptide into MS2 silk and then blending DOXMS2 with H2.1MS1, we gained additional control over Dox loading and release. Significantly higher Dox incorporation and lower drug release at pH 7.4 by the H2.1MS1:DOXMS2 particles compared with the H2.1MS1:MS2 spheres indicated that the functional peptide could prolong the drug entrapment within the carrier and induce more sustained drug release. Accordingly, the prolonged effect obtained in the present study by introducing the DOX affinity peptide could help to avoid the burst release of the drug into the bloodstream before the carrier reaches the tumor site.

### Cell binding assay of the silk spheres

**Fig. 6:**
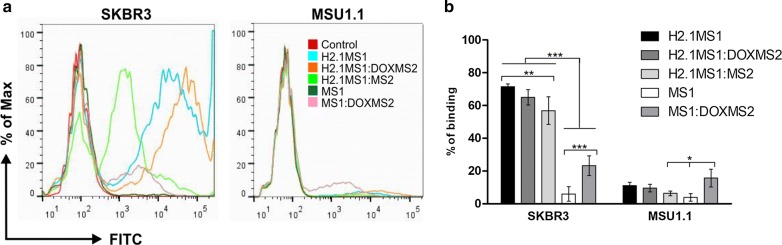
Cell binding assay of the silk spheres. Her2-positive cells (SKBR3) and Her2-negative cells (MSU1.1) were incubated with plain spheres composed of the control silk proteins MS1 and H2.1MS1 and with blended spheres (H2.1MS1:DOXMS2, H2.1MS1:MS2, and MS1:DOXMS2) prepared at an 8:2 weight ratio. **a** Representative graphs of the flow cytometry analysis. Nontreated cells were the control. **b** The mean percentage of silk sphere binding to cells (± SD) in three independent experiments is shown. *** indicates statistical significance with p < 0.001, **p < 0.01, and *p < 0.05

Fluorescently labeled functionalized and control silk spheres were incubated with cells, and cell binding by spheres was analyzed by flow cytometry. Figure 6a shows a representative sample from the flow cytometry analysis. The plain (H2.1MS1) and blended (H2.1MS1:DOXMS2 and H2.1MS1:MS2) silk particles functionalized with Her2-binding peptide showed significantly higher binding to Her2-overexpressing SKBR3 cancer cells compared with control particles (MS1 and MS1:DOXMS2) (Fig. 6b). Moreover, the binding of functionalized variant spheres to SKBR3 cells was significantly higher than the binding to Her2-negative cells (MSU1.1). These findings were consistent with our previous results confirming the specific binding of the functionalized spheres to Her2-overexpressing cells mediated by the H2.1 peptide [17].

Moreover, based on our previous results, we chose to mix the MS1 and MS2 silk types at a ratio of 8:2 because the H2.1MS1:H2.1MS2 spheres prepared under such conditions bound to Her2-positive cells at the same yield as the plain H2.1MS1 particles [21]. In this study, we showed lower cell binding by H2.1MS1:MS2 particles than that by plain H2.1MS1 spheres. This could have resulted from a decreased number of H2.1 peptides that was not fused to the MS2 silk. However, the presence of DOXMS2 silk within the blended spheres increased the particle binding compared to that of the blended spheres containing nonfunctionalized MS2 silk. Moreover, approximately 20% of the MS1:DOXMS2 variant spheres bound to cancer cells (SKBR3) and fibroblasts (MSU1.1) (Fig. 6b). On the other hand, the intensity of the fluorescent signals from cancer and control cells after exposure to the labeled MS1:DOXMS2 spheres was low, indicating the small number of particles bound per cell (Fig. 6a). In contrast, the analysis of the fluorescent signals from cancer cells after incubation with the labeled spheres containing H2.1 functionalized silk indicated that a higher number of spheres was bound per cell. The H2.1MS1:DOXMS2 spheres were the most numerously represented per cell (Fig. 6a). These data indicated that the DOX peptide could enhance the efficiency of H2.1MS1 particle binding to cells.

### Intracellular distribution of Dox

**Fig. 7:**
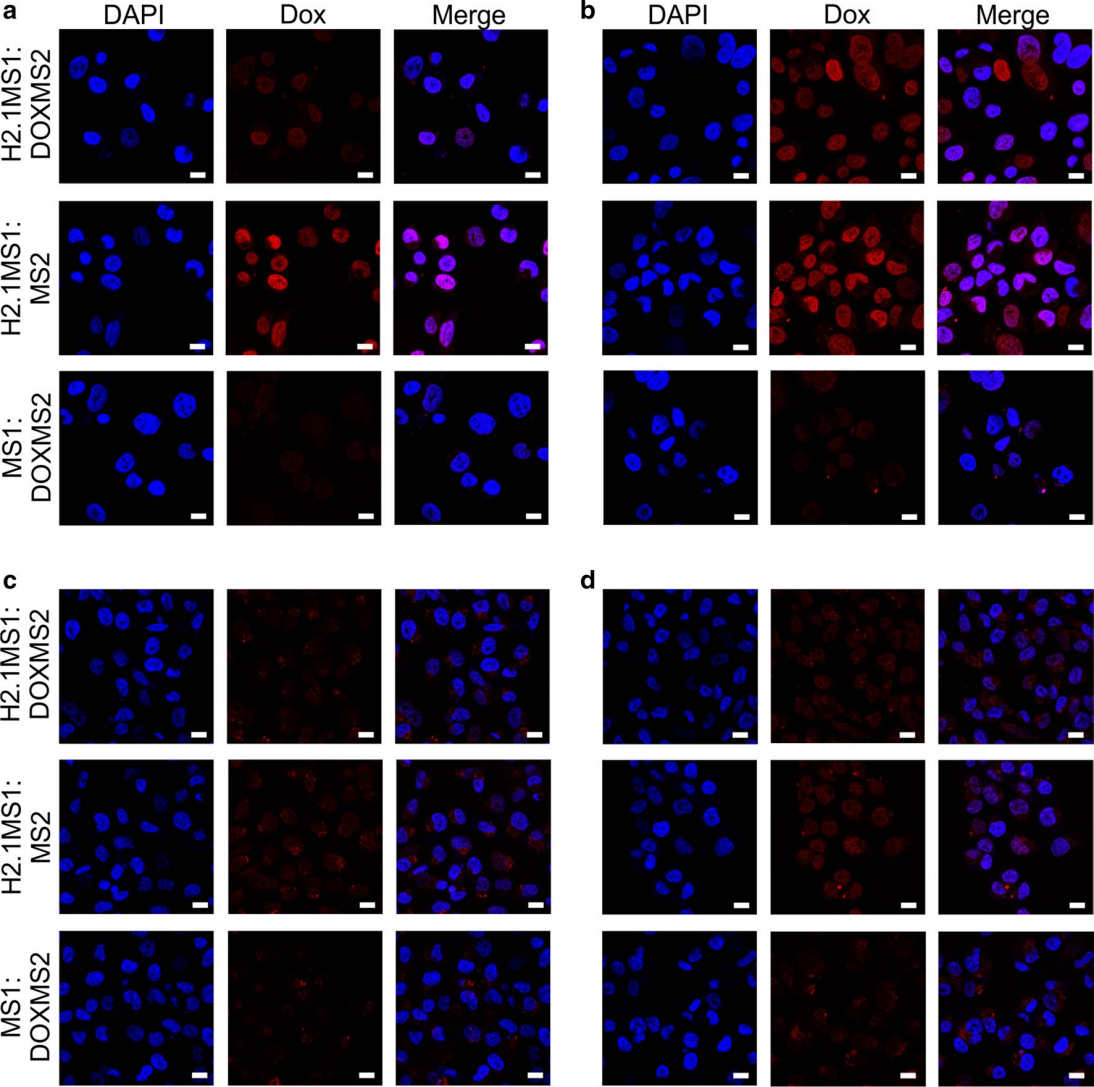
Confocal microscopy of the intracellular distribution of Dox-loaded silk spheres. (**a**, **b**) SKBR3 and (**c**, **d**) MSU1.1 cells were incubated at 37 °C for 15 (**a**, **c**) and 30 (**b**, **d**) min with Dox-loaded H2.1MS1:DOXMS2, H2.1MS1:MS2, and MS1:DOXMS2 spheres. DAPI: the nuclei stained with DAPI (blue); Dox: Dox-loaded spheres and Dox released from the spheres (red); merge: the colocalization of the nuclei and Dox. The scale bar represents 10 μm

The internalization of drugs into cancer cells is a prerequisite for their anticancer effects. The release of Dox from the spheres inside the cells was analyzed by confocal laser scanning microscopy utilizing Dox autofluorescence. The CLSM images showed that Dox accumulated in the nuclei of Her2-positive cells in a time-dependent manner (Fig. 7). As shown in Fig. 7a, a significant fraction of the Dox molecules accumulated in the nuclei of Her2-positive SKBR3 cells after 15 min of incubation with Dox-loaded H2.1MS1:MS2 spheres. After 30 min, a considerable amount of Dox was observed in the nuclei of cancer cells treated with Dox-loaded H2.1MS1:MS2 and H2.1MS1:DOXMS2 particles (Fig. 7b). Although the Dox accumulation in the nuclei after treatment with Dox-loaded H2.1MS1:MS2 particles was faster than that after exposure to Dox-loaded H2.1MS1:DOXMS2 particles, both sphere types effectively released the drug. Our previous studies indicated that spheres functionalized with H2.1 peptide were internalized via an endocytosis-dependent pathway [37]. Upon entering the cells, the H2.1-functionalized silk particles were trafficked to lysosomes where their degradation occurred [37]. The released Dox accumulated in the nucleus, which killed the cells [17]. Presumably, the same mechanism applied to the double-functionalized spheres. The DOX functional peptide did not impede the processing and final drug release of the silk spheres inside the cells.

In contrast, a negligible amount of Dox was detected in the nuclei of control MSU1.1 cells treated for 15 and 30 min with the blended sphere variants compared with that in the nuclei of SKBR3 cells (Fig. 7c and d). Furthermore, at the indicated time points, the Dox that had accumulated in the nuclei was hardly visible when it was released from nonfunctionalized MS1:DOXMS2 spheres in both Her2-positive and Her2-negative cells (Fig. 7). This relatively low nonspecific effect probably resulted from the residual release of the drug into the culture medium in which the cells were maintained. According to the results of Dox release, cells treated with silk spheres without DOX peptide released more drug into the medium; thus, its accumulation in nuclei was slightly higher than that observed in cells treated with drug-loaded DOX-functionalized spheres.

### Cytotoxicity of Dox delivered by functionalized spheres

**Fig. 8:**
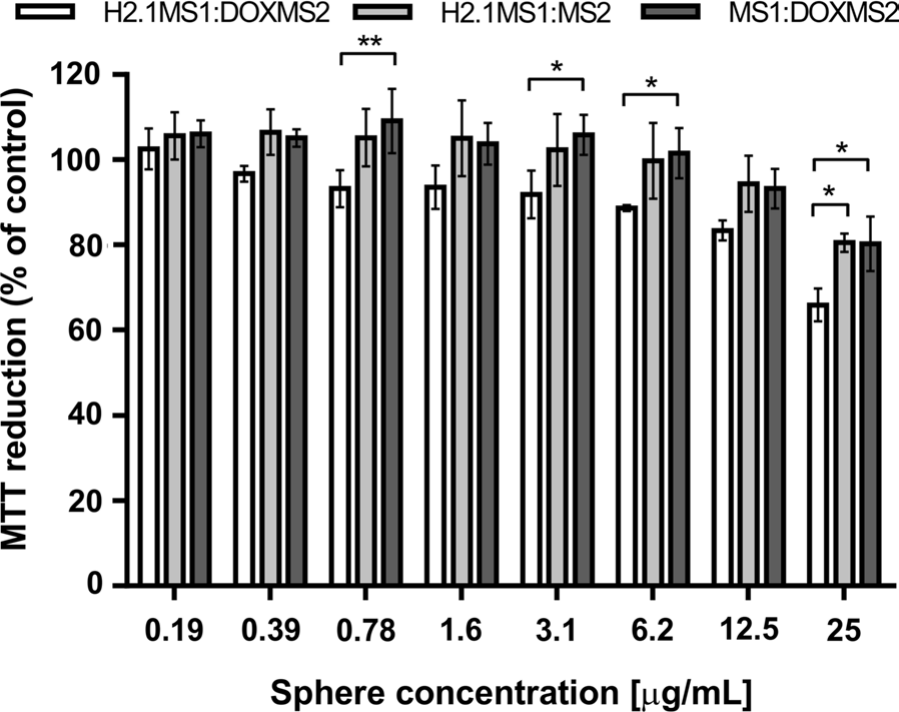
Cytotoxicity study performed using the MTT assay. SKBR3 cells were incubated in the presence of different concentrations of blended spheres for 15 min and then cultured for 72 h. The percentage of MTT reduction was calculated in reference to the nontreated control cells. The results demonstrate the mean and standard deviation of three independent experiments. * indicates statistical significance with p < 0.05; and **p < 0.01

In our previous studies, we analyzed the cytotoxicity of Dox delivered by H2.1MS1 functionalized spheres by incubating cells for 4 h with the drug-loaded spheres [17, 21]. We found significantly higher toxicity of Dox-loaded functionalized silk spheres towards target cells compared with that of control spheres and towards control cells [17, 21]. Because the intercellular distribution of Dox was different after 15 min of exposure to various silk spheres (Fig. 7a, b), we examined their cytotoxic effects at the same time point. The very short exposure of Her2(+) cells to Dox-loaded H2.1MS1:DOXMS2 spheres resulted in the highest cytotoxic effect compared with the impact of the other sphere variants (Fig. 8). The H2.1MS1:DOXMS2 spheres showed the highest Dox-loading capacity and binding per cell, which resulted in the highest cytotoxic effect compared to that of the other sphere variants. At least to some extent, the impact of DOX and the H2.1 peptides was additive in terms of overall drug delivery and cytotoxicity. However, this issue needs further examination.

There was no significant difference in toxicity towards SKBR3 cells in samples in which Dox was carried by spheres made of H2.1MS1:MS2 and MS1:DOXMS2 (Fig. 8). The nonspecific cytotoxic effect at the highest concentration of Dox-loaded MS1:DOXMS2 could have resulted from the residual release of the drug from spheres and/or nonspecific cell binding mediated by the DOX peptide. However, such a concentration of silk spheres in the body would be unattainable in vivo.

## Conclusions

The drug delivery system based on the use of silk material can be controlled by manipulating the composition of the silk spheres. We blended two silk types (MS1 and MS2) that were functionalized with peptides with different functions. The H2.1 peptide provides specificity, while the DOX peptide enables increased doxorubicin loading and decreases drug release in the bloodstream. Such a combination allows the formation of spheres that deliver a drug to target cells in a more controlled manner. Although the H2.1MS1 silk-containing spheres always indicated less spherical, more aggregated morphology [17, 21, 23], their systemic administration did not induce any toxic effects in mice (data not shown, manuscript in preparation). Thus, the silk blending strategy provides the opportunity to introduce other functionalization that can be useful to generate an optimal drug delivery system directed towards different cell types and/or other drugs.

## Materials and methods

### Construction of expression plasmids

The DOXMS2 gene was obtained by cloning DNA sequence encoding the DOX peptide into a pETNX-MS2 vector that was described in our previous work [20]. The pENTX-MS2 was digested with the NheI (Thermo Scientific, Waltham, MA) restriction enzyme before the ligation of the DOX oligonucleotide with the 3′ and 5′ cohesive ends complementary to those generated by the NheI and SpeI restriction enzymes, respectively. The oligonucleotide sequence encoding the DOX peptide were as follows: F: 5′-CTAGCCTGTGGAGCCCGTGGTATGGCGGTAGTTGGA-3′, R: 3′-CTAGTCCAACTACCGCCATACCACGGGCTCCACAGG-5′. The obtained plasmid was sequenced at the University of Adam Mickiewicz Core Facility in Poznan, Poland. The construction of the pETNX-MS1 and pETNX-H2.1-MS1 was described previously [17]. The T4 ligase was purchased from Promega (Madison, WI).

### Expression and purification of silk proteins

Plasmids pETNX-DOXMS2, pETNX-MS1, pETNX-MS2, and pETNX-H2.1-MS1 were introduced by transformation to the expression host—*Escherichia coli* BLR strain (DE3; Novagen, Madison, WI). The protein production was carried out in BioFlo415 (New Brunswick) fermentor, similarly to the method described previously [20]. The over-expression of proteins in the bacteria was induced with 1 mM isopropyl β-D-1 thiogalactopyranoside (IPTG; A&A Biotechnology, Gdansk, Poland). After 4 h, bacteria were collected by centrifugation (5 min, 9000×*g*) at room temperature. For the purification process, the formerly described thermal method (called 80/20) was applied [17]. The concentration of purified protein was determined spectrophotometrically at the 280 nm wavelength, referring to respective molecular weight (MW) and molar extinction coefficients. The MW was as follows: 39.54 kDa—MS1, 41.68 kDa—H2.1MS1, 46.72 kDa—MS2, 48.13 kDa—DOXMS2. The molar extinction coefficients were 22,350, 43,320, 44,700, 62,690 M^−1^ cm^−1^, respectively. The analysis of the quality of purified protein was performed by electrophoresis using 10% SDS-PAGE gel. Proteins were stained with colloidal Roti^®^ Blue Staining Solution (Carl Roth, Karlsruhe, Germany). For the cell binding assays, the bioengineered silks were first conjugated with the FITC fluorophore (Sigma, St. Louis, MO) according to the supplier’s protocol, and then they were used for sphere formation.

### Silk sphere formation by pipetting

The MS2 and DOXMS2 spheres were formed by mixing the respective protein with potassium phosphate buffer (Sigma-Aldrich, Saint Louis, MO) at a 1:10 volume ratio with a method similar to that described previously [20]. In brief, 100 µL of MS2 or DOXMS2 (at a concentration of 2.5 mg/mL) was added to 1000 µL of 2 M potassium phosphate buffer at pH 8.0 and mixed by pipetting. The obtained spheres were then incubated overnight at room temperature and then dialyzed against deionized water for 3 days using a ZelluTrans dialysis membrane with an MWCO of 12–14 kDa (Carl Roth, Karlsruhe, Germany). After dialysis, the spheres were collected by centrifugation at 23,000×*g* for 1 h at room temperature and resuspended in ultra-pure water.

### Silk sphere formation by micromixing

The H2.1MS1:DOXMS2, MS1:DOXMS2, and H2.1MS1:MS2 spheres were formed through mixing proteins with potassium phosphate buffer at a 1:10 volume ratio using a micromixing system similar to the method described previously [23]. In brief, before the production process, the corresponding proteins at a concentration of 0.5 mg/mL were mixed at a ratio of 8:2 (v/v), respectively. Then, the blended silk spheres were formed by mixing the proteins with 2 M potassium phosphate buffer, pH 8, by using the neMESYS high-pressure syringe pump system (Cetoni GmbH, Korbußen, Germany) under the control of neMESYS UserInterface software (Cetoni GmbH, Korbuβen, Germany). The proteins and potassium phosphate were mixed at a ratio of 1:10 by setting the flow of the protein to 10 µL/s and that of potassium phosphate to 100 µL/s. For the production process, tubes with a diameter of 250 µm and a T-shaped mixing element with a circular mixing zone with a diameter of 150 µm were used. Next, the spheres were incubated overnight at room temperature in the presence of potassium phosphate buffer and then dialyzed against deionized water and collected as described above. The sphere concentration was determined gravimetrically.

### Morphology of the silk spheres by scanning electron microscopy (SEM)

The spheres’ morphology was analyzed using a scanning electron microscope (SEM). Silk spheres suspended in the deionized water were dropped onto the cover glass (Nunc, Naperville, IL) and left to air-dry overnight. The samples were sputtered with a gold layer using Quorum Sputter Coater Q150T ES (Quorum Technologies, Ringmer, UK) and analyzed under JEOL JSM-7001F (JEOL. Ltd, Tokyo, Japan) field emission scanning electron microscope at 15 kV accelerating voltage. The spheres’ diameter was measured with ImageJ 1.51 K software. The mean size of the particles was calculated based on the measurement of the diameter of 30 different spheres on three independent photographs.

### Loading and release of doxorubicin from silk spheres

The MS2 and DOXMS2 particles and the spheres made of the H2.1MS1:MS2, H2.1MS1:DOXMS2, and MS1:DOXMS2 blends were loaded with doxorubicin (Adriamycin, Pfizer Inc., New York City, NY) using the postloading method as described previously [20]. Briefly, 250 µg of spheres was suspended in 250 µL of PBS, mixed with 50 µL of 2 mg/mL doxorubicin, and incubated at room temperature under continuous shaking. After overnight incubation, the spheres were centrifuged for 15 min at 10,000×*g*, and the absorbance of the supernatant was measured at 508 nm to determine the drug concentration. The quantification of the drug was based on a standard calibration curve for doxorubicin. The encapsulation efficiency was determined using the following equation: (amount of drug-loaded)/(amount of drug initially added) × 100%.

For release study, the doxorubicin-loaded spheres were incubated in 1 mL of phosphate buffer solution at pH 7.4, 6 and 4.5 at 37 °C with constant shaking. At indicated time points, the spheres were centrifuged for 15 min at 10,000×*g*, and then the supernatant was collected and replaced with fresh PBS of proper pH values. The amount of released drug was determined spectrophotometrically, as specified above.

### Cell culture

In the study, human breast cancer cell line SKBR-3 (ATCC, Manassas, VA) and human fibroblast cell line MSU1.1 (obtained thanks to the courtesy of professor C. Kieda, (CBM, CNRS, Orleans, France)) were used. Cells were cultured in Dulbecco’s Modified Eagle Medium (DMEM; PAA Laboratories GmbH, Pasching, Austria) supplemented with 10% fetal bovine serum (PAA Laboratories GmbH,Pasching, Austria) and 80 μg/mL gentamycin (KRKA, NovoMesto, Slovenia) at 37 °C in a humidified atmosphere enriched in 5% CO_2_.

### Cell binding assay of silk spheres

The SKBR3 and MSU1.1 cells were washed with PBS/0.5% BSA and detached with non-enzymatic cell dissociation solution (Sigma, St. Louis, MO) according to the manufacturer’s protocol. Next, 30 μL of FITC-labeled spheres at a final concentration of 10 μg/mL was added to 1 × 10^5^ of cells and incubated for 1 h at 4 °C in the dark. The binding of the spheres to the cells was analyzed using an FL2 channel on a FACSAria flow cytometer (BD Biosciences Pharmingen, San Jose, CA) and FACSDiva (v6.1.2) software. Three independent experiments were performed. The representative graphs of the flow cytometry analysis were prepared using FlowJo V10 software (Tree Star, Ashland, OR).

### Intracellular distribution of Dox

SKBR3 and MSU1.1 cells (1 × 10^5^ cells/well) were plated on 8-well Lab-Tek chambered coverslips (Nunc, Naperville, IL) and cultured for 24 h. Next, 10 μg/mL of Dox-loaded H2.1MS1:MS2, H2.1MS1:DOXMS2, and MS1:DOXMS2 spheres were added to the cells, which were incubated at 37 °C for 15 or 30 min. After washing with PBS, the cells were fixed with 4% paraformaldehyde (PFA; Electron Microscopy Sciences, Hatfield, PA). Subsequently, the cells were washed with PBS and immersed in Fluoroshield mounting medium with DAPI (Sigma, St. Louis, MO) and then analyzed under an Olympus FV1000 scanning confocal microscope (Shinjuku, Tokyo, Japan) connected to a blue laser diode and an argon laser. Image acquisition and analysis were performed with a 60× objective, a 1.4 N.A. oil immersion lens, and FLUOVIEW Viewer software, ver. 4.1. The nuclei were visualized using 350 nm excitation and 440–480 nm emission wavelengths. To visualize the Dox-loaded spheres and the Dox released from the spheres, an excitation wavelength of 488 nm and an emission wavelength of 570–610 nm were used.

### Cytotoxicity study

A total of 2.5 × 10^4^ SKBR3 cells per well were seeded into a 96-well plate and incubated overnight at 37 °C in a humidified atmosphere containing 5% CO_2_. The next day, different concentrations of blended silk spheres loaded with Dox were added to the cell cultures. As a negative control, cells without spheres were used. After 15 min of incubation, cells were washed twice with PBS, and fresh medium was added. After 3 days of incubation, 50 μL (5 mg/mL) of MTT reagent (3-(4,5-dimethylthiazol-2-yl)-2,5-diphenyl tetrazolium bromide; Sigma, St. Louis, MO) was added to each well, and an additional incubation of 4 h was performed. Next, the medium was removed, and 200 μL of dimethyl sulfoxide (Sigma, St. Louis, MO) was added to dissolve the insoluble formazan. The absorbance was measured at a wavelength of 560 nm using a Victor X3 Multimode Plate Reader (PerkinElmer, Waltham, MA) controlled by PerkinElmer 2030 Workstation software (PerkinElmer, Waltham, MA). The relative cell viability (%) compared to that of the negative control was calculated using the following equation: test sample/negative control × 100%. The experiment was repeated three times in triplicate.

### Statistics

The statistical significance of the differences between sphere groups was calculated using a one-way ANOVA test with Bonferroni posthoc correction. The differences between groups were considered significant if the p-value < 0.05.

## Data Availability

All data generated or analyzed during this study are included in this published article.
